# Modeling and Analysis of Micro-Spacecraft Attitude Sensing with Gyrowheel

**DOI:** 10.3390/s16081321

**Published:** 2016-08-19

**Authors:** Xiaokun Liu, Hui Zhao, Yu Yao, Fenghua He

**Affiliations:** School of Astronautics, Harbin Institute of Technology, No. 2 Yikuang Street, Nangang District, Harbin 150080, China; zhaohui@hit.edu.cn (H.Z.); yaoyu@hit.edu.cn (Y.Y.); hefenghua@hit.edu.cn (F.H.)

**Keywords:** gyrowheel, micro-spacecraft angular rate sensing, real-time Lyapunov linearization, complex quantity, static measurement, dynamic measurement

## Abstract

This paper proposes two kinds of approaches of angular rate sensing for micro-spacecraft with a gyrowheel (GW), which can combine attitude sensing with attitude control into one single device to achieve a compact micro-spacecraft design. In this implementation, during the three-dimensional attitude control torques being produced, two-dimensional spacecraft angular rates can be sensed from the signals of the GW sensors, such as the currents of the torque coils, the tilt angles of the rotor, the motor rotation, etc. This paper focuses on the problems of the angular rate sensing with the GW at large tilt angles of the rotor. For this purpose, a novel real-time linearization approach based on Lyapunov’s linearization theory is proposed, and a GW linearized measurement model at arbitrary tilt angles of the rotor is derived. Furthermore, by representing the two-dimensional rotor tilt angles and tilt control torques as complex quantities and separating the twice periodic terms about the motor spin speed, the linearized measurement model at smaller tilt angles of the rotor is given and simplified. According to the respective characteristics, the application schemes of the two measurement models are analyzed from the engineering perspective. Finally, the simulation results are presented to demonstrate the effectiveness of the proposed strategy.

## 1. Introduction

Presently, more and more researchers are focusing on the related technologies of micro-spacecraft [1,2,3]. For micro-spacecraft, the attitude control system (ACS) is one of the major contributors to the mass, volume, power and cost [4]. It will be of great significance for the development of micro-spacecraft if the realization of spacecraft angular rate sensing and the output of three-dimensional control torques can be simultaneously conducted in one instrument [5,6]. For this purpose, some innovative actuators like a variable speed control moment gyroscope (VSCMG) [7], a tilted wheel [8,9], and so on [10], which also have potential to realize the function of the sensors in principle, have been developed. Due to the existence of nonlinear friction from the support structure of some actuators, it is complicated to realize the angular rate sensing for them. However, besides that, according to the difference of the gyro rotor support and combining the advantages of the actuator-CMG [11] with the ones of the sensor dynamically-tuned gyroscope (DTG) [12], the integrated devices can be roughly divided into two categories: the magnetically-suspended double-gimbal control moment gyroscope (MSDGCMG) [13] and the integrated device-based flexible gimbal support structure represented by the gyrowheel (GW) [14]. The former one supports the rotor by active magnetic bearings (AMBs). The latter, GW, is developed based on the principle of DTG by Bristol Aerospace Company for the Canadian Space Agency’s SCISAT-1 Scientific in 2003 [15], and the GW rotor is supported by crossed torsion springs and a gimbal. The MSDGCMG implements the functions of two degrees of freedom (DOF) torque output and two-axis angular rate sensing through working in two different operation modes [16]. However, due to the complicated structure, high mass and large volume, the application of MSDGCMG in micro-spacecraft is seriously restricted. Unlike MSDGCMG, the GW not only can achieve three-dimensional torque output and two-axis angular rate sensing at the same time, but also has the advantage of being light weight and low power, which brings the hope that the GW will have promising application prospects. Therefore, it makes sense to develop the method of the angular rate sensing with GW as an actuator and a redundant measurement device simultaneously.

GW and DTG have similar structures. However, DTG always operates in the fixed tuned speed, and the spin axis of the rotor cannot tilt in radial directions theoretically. However, GW is almost one thousand times greater than DTG in mass, moment of inertia and angular momentum [17]. To realize torque outputs along the radial directions of the rotor, the tilt range of the spin axis of the rotor in GW is significantly increased up to 7° [18]. To realize torque output along the spin direction, the operating speed of the rotor is always kept as time varying. Obviously, GW has more complex dynamical characteristics than DTG. Thus, it is more complicated for GW to realize the spacecraft angular rate sensing while three-dimensional control torques are outputted.

In order to realize the angular rate sensing of the carrier by GW, Dr. Own at Carleton University in Canada realized his work by linearizing the equations of motion of GW [19] at zero tilt angles of the rotor. However, a higher measurement accuracy can be obtained only if the tilt angles of the rotor spin axis are limited to a very small range. This disadvantage imposes strict restrictions on the capacity of the GW output torque, while a higher measurement accuracy is needed. Although Jeffrey M. Hall at Carleton University improved the measurement accuracy of the two-dimensional rate sensing through the ground calibration for the GW [20], the accuracy loss caused by linearization at zero tilt angles cannot be easily compensated at larger tilt angles. Moreover, before the linearization of the equations of motion of GW, an assumption that motor spin speed was always kept constant was made by Dr. Own, which means that the spacecraft angular rates cannot be accurately measured while the control torques along the motor spin axis are outputted by adjusting the motor spin speed. Liu proposed the nonlinear algebraic measurement method of the spacecraft angular rates with the GW supported by the torsion springs through analyzing the full GW dynamics equations [21], but it is hard to calibrate the compensable gyroscopic drift for this nonlinear algebraic measurement method [22], which directly leads to the inaccuracy of the measurement results. Liu also proposed a dynamic measurement method by establishing GW nonlinear state equations [23]. However, it is also difficult to compensate the modeling errors due to the nonlinear form of the state equations.

To overcome the aforementioned drawbacks of the spacecraft angular rate sensing with GW at present, the reminder of this paper is divided into five sections: In Section 2, the GW is succinctly described, and then, its dynamics equations expressed by case coordinates are developed by the Lagrange equations of the second kind. In Section 3, the GW real-time Lyapunov linearization measurement model at arbitrary operating points is established based on Lyapunov’s linearization theory. Especially, by the complex quantity method, the GW complex differential equations within small tilt angles of the nominal position are derived. The small tilt measurement model is established and simplified by ignoring the twice periodic components about the motor spin rate. In Section 4, according to the characteristics of these two proposed measurement models, the application schemes are analyzed from the perspectives of the static measurement and dynamic measurement. In Section 5, simulations are performed to illustrate the validity of the proposed linearization measurement models and their application schemes in this paper. Finally, we draw the conclusions in Section 6.

## 2. Descriptions of Gyrowheel

### 2.1. Gyrowheel Physical Structure

**Figure 1:**
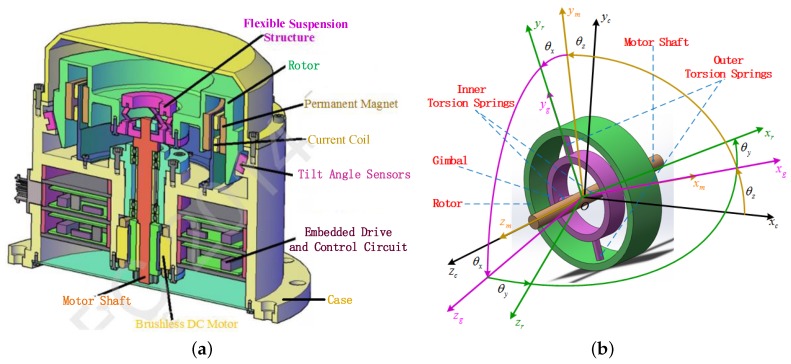
Gyrowheel physical structure. (**a**) Schematic diagram of a gyrowheel system; (**b**) simplified gyrowheel structure diagram.

The computer-aided design diagram and simplified structure of the GW are shown in Figure 1a,b, respectively. The GW system mainly consists of the following subassemblies: case, motor, flexible suspension structure, flywheel rotor, torquer consisting of current coil and permanent magnet and tilt sensor. Among them, the case is fixed on the carrier, such as spacecraft. The flexible suspension structure is made up of gimbal, inner and outer torsion springs, as shown in Figure 1b; the gimbal is connected to the motor shaft by a pair of inner torsion springs, and the rotor is connected with the gimbal by a pair of outer torsion springs. The rotor driven by the brushless DC motor rotates in a high time-varying speed. Thus, the torque along the spin direction of the rotor can be generated by adjusting the motor speed. Two pairs of torquers perpendicular to each other can provide two-dimensional tilt control torques to make the spin axis of the rotor tilt along the radial directions. Due to the existence of the angular momentum, the radial control torques can be outputted based on the CMG principle. The tilt sensors are designed to measure the tilt angles of the rotor relative to the case. The special physical structure of GW determines that the device can realize the functions of the sensor, like DTG, and the actuator, like VSCMG, at the same time.

### 2.2. Equations of Motion for an Idealized Gyrowheel

**Figure 2:**
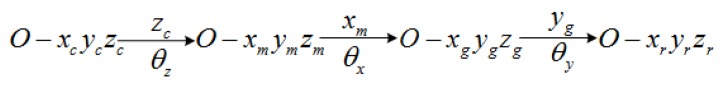
Relationship between the body frames and the generalized coordinates.

Four body frames and three generalized coordinates are given in Figure 1b for deriving GW dynamics equations using Lagrange’s method. The four body frames are the case frame ($F_{0}$:*O*-$x_{c}$$y_{c}$$z_{c}$), the motor body frame ($F_{1}$:*O*-$x_{m}$$y_{m}$$z_{m}$), the gimbal body frame ($F_{2}$:*O*-$x_{g}$$y_{g}$$z_{g}$) and the rotor body frame ($F_{3}$:*O*-$x_{r}$$y_{r}$$z_{r}$), respectively. The coordinates ($\theta_{x},\theta_{y},\theta_{z}$) are the defined generalized coordinates, where $\theta_{x}$ and $\theta_{y}$ represent the rotation angles of the inner and outer torsion springs, respectively, and $\theta_{z}$ represents the spin angle of the motor shaft. The relationship between these four body frames and the generalized coordinates can be further expressed by Figure 2.

Assume that the angular velocity of the GW case fixed on the spacecraft, with respect to inertial space in the case frame $F_{0}$, is $\omega_{b}$ = ${\left[\omega_{bx}\;\omega_{by}\;\omega_{bz}\right]}^{T}$.

Based on the relationship in Figure 2, the angular velocity of the motor shaft $\omega_{m}$ in the motor body frame $F_{1}$ is described as the following equation:

(1) $$\omega_{m}=\left[\begin{array}{c} \omega_{mx}\\ \omega_{my}\\ \omega_{mz} \end{array}\right]=\left[\begin{array}{c} 0\\ 0\\ {\dot{\theta }}_{z} \end{array}\right]+{\left(\theta_{z}\right)}_{z}\cdot \omega_{b}=\left[\begin{array}{c} \omega_{bx}C_{\theta_{z}}+\omega_{by}S_{\theta_{z}}\\ -\omega_{bx}S_{\theta_{z}}+\omega_{by}C_{\theta_{z}}\\ {\dot{\theta }}_{z}+\omega_{bz} \end{array}\right]$$

where $S_{\theta_{i}}=\sin\theta_{i}$, $C_{\theta_{i}}=\cos\theta_{i},i=x,y,z$ and ${\left(\theta_{i}\right)}_{i},i=x,y,z$ represents the rotation matrix of $\theta_{i}$ about the $z_{m}$-axis, $x_{g}$-axis and $y_{g}$-axis, respectively.

Similarly, the angular velocity of the gimbal can be obtained by rotating the motor shaft speed, ${\dot{\theta }}_{z}$, into the gimbal body frame and adding the rate about the *x*-axis, so we have:

(2) $$\omega_{g}=\left[\begin{array}{c} \omega_{gx}\\ \omega_{gy}\\ \omega_{gz} \end{array}\right]=\left[\begin{array}{c} {\dot{\theta }}_{x}\\ 0\\ 0 \end{array}\right]+{\left(\theta_{x}\right)}_{x}\cdot \omega_{m}=\left[\begin{array}{c} {\dot{\theta }}_{x}+\omega_{bx}C_{\theta_{z}}+\omega_{by}S_{\theta_{z}}\\ -\omega_{bx}C_{\theta_{x}}S_{\theta_{z}}+\omega_{by}C_{\theta_{x}}C_{\theta_{z}}+\left({\dot{\theta }}_{z}+\omega_{bz}\right)S_{\theta_{x}}\\ \omega_{bx}S_{\theta_{x}}S_{\theta_{z}}-\omega_{by}S_{\theta_{x}}C_{\theta_{z}}+\left({\dot{\theta }}_{z}+\omega_{bz}\right)C_{\theta_{x}} \end{array}\right]$$

Finally, rotating the gimbal angular velocities into the rotor body frame and then adding the rate about the *y*-axis, the angular velocities of the rotor are given as follows:

(3) $$\begin{array}{cc} \omega_{r} & =\left[\begin{array}{c} \omega_{rx}\\ \omega_{ry}\\ \omega_{rz} \end{array}\right]=\left[\begin{array}{c} 0\\ {\dot{\theta }}_{y}\\ 0 \end{array}\right]+{\left(\theta_{y}\right)}_{y}\cdot \omega_{g}\\ & =\left[\begin{array}{c} {\dot{\theta }}_{x}C_{\theta_{y}}-{\dot{\theta }}_{z}C_{\theta_{x}}S_{\theta_{y}}+\left(C_{\theta_{y}}C_{\theta_{z}}-S_{\theta_{x}}S_{\theta_{y}}S_{\theta_{z}}\right)\omega_{bx}+\left(C_{\theta_{y}}S_{\theta_{z}}+S_{\theta_{x}}S_{\theta_{y}}C_{\theta_{z}}\right)\omega_{by}-C_{\theta_{x}}S_{\theta_{y}}\omega_{bz}\\ {\dot{\theta }}_{z}S_{\theta_{x}}+{\dot{\theta }}_{y}-C_{\theta_{x}}S_{\theta_{z}}\omega_{bx}+C_{\theta_{x}}C_{\theta_{z}}\omega_{by}+S_{\theta_{x}}\omega_{bz}\\ {\dot{\theta }}_{x}S_{\theta_{y}}+{\dot{\theta }}_{z}C_{\theta_{x}}C_{\theta_{y}}+\left(S_{\theta_{y}}C_{\theta_{z}}+S_{\theta_{x}}C_{\theta_{y}}S_{\theta_{z}}\right)\omega_{bx}+\left(S_{\theta_{y}}S_{\theta_{z}}-S_{\theta_{x}}C_{\theta_{y}}C_{\theta_{z}}\right)\omega_{by}+C_{\theta_{x}}C_{\theta_{y}}\omega_{bz} \end{array}\right] \end{array}$$

According to the calculated angular rates of the above different GW bodies, the kinetic energy *T* of the GW system can be expressed as the generalized rotation speed quadratic forms:

(4) $$T=\frac{1}{2}\left(\sum_{i=x,y,z}I_{mi}\omega_{mi}^{2}+\sum_{i=x,y,z}I_{gi}\omega_{gi}^{2}+\sum_{i=x,y,z}I_{ri}\omega_{ri}^{2}\right)$$

where $I_{ri}$ and $I_{gi}$,i=x,y,z are the moments of inertia of the rotor and gimbal along the corresponding axes, respectively.

Furthermore, the potential energy *V* is the sum of the potential energy of the inner and outer torsion deformation, which is given by:

(5) $$V=k_{x}\theta_{x}^{2}+k_{y}\theta_{y}^{2}$$

Forming the Lagrangian function L=T-V and applying Lagrange’s equations over $\theta_{x}$ and $\theta_{y}$ yield the GW dynamics equations along the transverse axes as follows:

(6) $$\begin{array}{cc} I_{1}\cdot {\ddot{\theta }}_{x} & =-c_{x}{\dot{\theta }}_{x}-k_{x}\theta_{x}-\frac{1}{2}I_{2}S_{2\theta_{x}}\cdot {{\dot{\theta }}_{z}}^{2}-I_{3}S_{2\theta_{y}}\cdot {\dot{\theta }}_{x}{\dot{\theta }}_{y}\\ & -\left(I_{3}C_{2\theta_{y}}-I_{ry}\right)C_{\theta_{x}}\cdot {\dot{\theta }}_{y}{\dot{\theta }}_{z}+T_{gx}\\ & +B_{1}\left(\theta \right)\omega_{bx}+B_{2}\left(\theta \right)\omega_{by}+B_{3}\left(\theta \right){\dot{\omega }}_{bx}+B_{4}\left(\theta \right){\dot{\omega }}_{by}\\ & +B_{5}\left(\theta \right)\omega_{bx}^{2}+B_{6}\left(\theta \right)\omega_{by}^{2}+B_{7}\left(\theta \right)\omega_{by}^{2}\\ I_{ry}\cdot {\ddot{\theta }}_{y} & =-c_{y}{\dot{\theta }}_{y}-k_{y}\theta_{y}-\frac{1}{2}I_{3}C_{\theta_{x}}^{2}S_{2\theta_{y}}\cdot {\dot{\theta }}_{z}^{2}+\frac{1}{2}I_{3}S_{2\theta_{y}}\cdot {\dot{\theta }}_{x}^{2}\\ & +\left(I_{3}C_{2\theta_{y}}-I_{ry}\right)C_{\theta_{x}}\cdot {\dot{\theta }}_{x}{\dot{\theta }}_{z}+T_{gy}\\ & +D_{1}\left(\theta \right)\omega_{bx}+D_{2}\left(\theta \right)\omega_{by}+D_{3}\left(\theta \right){\dot{\omega }}_{bx}+D_{4}\left(\theta \right){\dot{\omega }}_{by}\\ & +D_{5}\left(\theta \right)\omega_{bx}^{2}+D_{6}\left(\theta \right)\omega_{by}^{2}+D_{7}\left(\theta \right)\omega_{by}^{2} \end{array}$$

where $I_{1}=I_{gx}+I_{rx}C_{\theta_{y}}^{2}+I_{rz}S_{\theta_{y}}^{2},I_{2}=I_{gz}-I_{gy}-I_{ry}+I_{rx}S_{\theta_{y}}^{2}+I_{rz}C_{\theta_{y}}^{2},I_{3}=I_{rz}-I_{rx}$ and $B_{i}$, $D_{i}$, i=1,2⋯,7 are nonlinear coefficients in terms of the spacecraft angular rates ($\omega_{bx},\omega_{by}$) as follows:

$$\begin{array}{cc} B_{1}= & \left[-I_{re}S_{\theta x}S_{2\theta_{y}}C_{\theta_{z}}+I_{1}S_{\theta_{z}}+I_{2}\cos2x_{1}S_{\theta_{z}}\right]{\dot{\theta }}_{z}\\ & -\left[I_{re}\left(S_{2\theta_{y}}C_{\theta_{z}}+S_{\theta x}C_{2\theta_{y}}S_{\theta_{z}}\right)-I_{ry}S_{\theta x}S_{\theta_{z}}\right]{\dot{\theta }}_{y}\\ B_{2}= & -\left[I_{re}S_{\theta x}S_{2\theta_{y}}S_{\theta_{z}}+I_{1}C_{\theta_{z}}+I_{2}C_{2\theta_{y}}C_{\theta_{z}}\right]{\dot{\theta }}_{z}\\ & -\left[I_{re}\left(S_{2\theta_{y}}S_{\theta_{z}}-S_{\theta x}C_{2\theta_{y}}C_{\theta_{z}}\right)+I_{ry}S_{\theta x}C_{\theta_{z}}\right]{\dot{\theta }}_{y}\\ B_{3}= & -I_{1}C_{\theta_{z}}-\frac{1}{2}I_{re}S_{\theta x}S_{2\theta_{y}}S_{\theta_{z}}\\ B_{4}= & -I_{1}S_{\theta_{z}}+\frac{1}{2}I_{re}S_{\theta x}S_{2\theta_{y}}C_{\theta_{z}}\\ B_{5}= & \frac{1}{2}I_{2}S_{\theta_{z}}^{2}S_{2\theta_{x}}+\frac{1}{4}I_{re}C_{\theta x}S_{2\theta_{y}}S_{2\theta_{z}}\\ B_{6}= & \frac{1}{2}I_{2}C_{\theta_{z}}^{2}S_{2\theta_{x}}-\frac{1}{4}I_{re}C_{\theta x}S_{2\theta_{y}}S_{2\theta_{z}}\\ B_{7}= & -\frac{1}{2}I_{2}S_{2\theta_{z}}S_{2\theta_{x}}-\frac{1}{2}I_{re}C_{\theta x}S_{2\theta_{y}}C_{2\theta_{z}} \end{array}$$

$$\begin{array}{cc} & D_{1}=\left[I_{re}\left(S_{2\theta_{y}}C_{\theta_{z}}+S_{\theta_{x}}C_{2\theta_{y}}S_{\theta_{z}}\right)-I_{ry}S_{\theta_{x}}S_{\theta_{z}}\right]{\dot{\theta }}_{x}\\ & +\left[I_{re}\left(C_{\theta_{x}}C_{2\theta_{y}}C_{\theta_{z}}-\frac{1}{2}S_{2\theta_{x}}S_{2\theta_{y}}S_{\theta_{z}}\right)+I_{ry}C_{\theta_{x}}C_{\theta_{z}}\right]{\dot{\theta }}_{z}\\ & D_{2}=\left[I_{re}\left(S_{2\theta_{y}}S_{\theta_{z}}-S_{\theta_{x}}C_{2\theta_{y}}C_{\theta_{z}}\right)+I_{ry}S_{\theta_{x}}C_{\theta_{z}}\right]{\dot{\theta }}_{x}\\ & +\left[I_{re}\left(\frac{1}{2}S_{2\theta_{x}}S_{2\theta_{y}}C_{\theta_{z}}+C_{\theta_{x}}C_{2\theta_{y}}S_{\theta_{z}}\right)+I_{ry}C_{\theta_{x}}S_{\theta_{z}}\right]{\dot{\theta }}_{z}\\ & D_{3}=I_{ry}C_{\theta_{x}}S_{\theta_{z}}\;D_{4}=-I_{ry}C_{\theta_{x}}C_{\theta_{z}}\\ & D_{5}=\frac{1}{2}I_{re}\left[S_{\theta_{x}}C_{2\theta_{y}}S_{2\theta_{z}}+S_{2\theta_{y}}\left(C_{\theta_{z}}^{2}-S_{\theta_{x}}^{2}S_{\theta_{z}}^{2}\right)\right]\\ & D_{6}=-\frac{1}{2}I_{re}\left[S_{\theta_{x}}C_{2\theta_{y}}S_{2\theta_{z}}-S_{2\theta_{y}}\left(S_{\theta_{z}}^{2}-S_{\theta_{x}}^{2}C_{\theta_{z}}^{2}\right)\right]\\ & D_{7}=\frac{1}{2}I_{re}\left(S_{\theta_{x}}^{2}S_{2\theta_{y}}S_{2\theta_{z}}-2S_{\theta_{x}}C_{2\theta_{y}}C_{2\theta_{z}}+S_{2\theta_{y}}S_{2\theta_{z}}\right) \end{array}$$

Actually, the generalized coordinates $\left(\theta_{x},\theta_{y}\right)$ in Equation (6) represent the rotation angles of the inner and outer torsion springs, which cannot be indirectly measured. Therefore, another set of coordinates $\left(\phi_{x},\phi_{y}\right)$ named “case coordinates” should be defined in the case frame $F_{0}$, and the case coordinates $\left(\phi_{x},\phi_{y}\right)$ physically represent the tilt angles of the rotor along the $Ox_{c}$-axis and $Oy_{c}$-axis, which can be measured by the tilt sensors of GW. Then, the relationships between the generalized coordinates and the case coordinates should be established. The detailed derivation will not be shown in this paper, and the relationships given in the development of DTG are as follows:

(7) $$\begin{array}{cc} \theta_{x} & =\phi_{x}C_{\theta_{z}}+\phi_{y}S_{\theta_{z}}\\ \theta_{y} & =-\phi_{x}S_{\theta_{z}}+\phi_{y}C_{\theta_{z}} \end{array}$$

The variables (${\dot{\theta }}_{x},{\dot{\theta }}_{y}$) and (${\ddot{\theta }}_{x},{\ddot{\theta }}_{y}$) in Equation (6) can be calculated by taking the first and second derivatives of Equation (7), then we have:

(8) $$\begin{array}{cc} {\dot{\theta }}_{x} & ={\dot{\phi }}_{x}C_{\theta_{z}}-\phi_{x}{\dot{\theta }}_{z}S_{\theta_{z}}+{\dot{\phi }}_{y}S_{\theta_{z}}+\phi_{y}{\dot{\theta }}_{z}C_{\theta_{z}}\\ {\dot{\theta }}_{y} & =-{\dot{\phi }}_{x}S_{\theta_{z}}-\phi_{x}{\dot{\theta }}_{z}C_{\theta_{z}}+{\dot{\phi }}_{y}C_{\theta_{z}}-\phi_{y}{\dot{\theta }}_{z}S_{\theta_{z}} \end{array}$$

(9) $$\begin{array}{cc} {\ddot{\theta }}_{x}= & {\ddot{\phi }}_{x}C_{\theta_{z}}-{\dot{\phi }}_{x}{\dot{\theta }}_{z}S_{\theta_{z}}-\left({\dot{\phi }}_{x}{\dot{\theta }}_{z}+\phi_{x}{\ddot{\theta }}_{z}\right)S_{\theta_{z}}-\phi_{x}{\dot{\theta }}_{z}^{2}C_{\theta_{z}}\\ & +{\ddot{\phi }}_{y}S_{\theta_{z}}+{\dot{\phi }}_{y}{\dot{\theta }}_{z}C_{\theta_{z}}+\left({\dot{\phi }}_{y}{\dot{\theta }}_{z}+\phi_{y}{\ddot{\theta }}_{z}\right)C_{\theta_{z}}-\phi_{y}{\dot{\theta }}_{z}^{2}S_{\theta_{z}}\\ {\ddot{\theta }}_{y}= & -{\ddot{\phi }}_{x}S_{\theta_{z}}-{\dot{\phi }}_{x}{\dot{\theta }}_{z}C_{\theta_{z}}-\left({\dot{\phi }}_{x}{\dot{\theta }}_{z}+\phi_{x}{\ddot{\theta }}_{z}\right)C_{\theta_{z}}+\phi_{x}{\dot{\theta }}_{z}^{2}S_{\theta_{z}}\\ & +{\ddot{\phi }}_{y}C_{\theta_{z}}-{\dot{\phi }}_{y}{\dot{\theta }}_{z}S_{\theta_{z}}-\left({\dot{\phi }}_{y}{\dot{\theta }}_{z}+\phi_{y}{\ddot{\theta }}_{z}\right)S_{\theta_{z}}-\phi_{y}{\dot{\theta }}_{z}^{2}C_{\theta_{z}} \end{array}$$

At this point, assume the rotor transverse inertias are equal to each other and set as the value $I_{rt}$. Similarly, the gimbal transverse inertias are also equal to each other and set as the values $I_{gt}$, that is,

(10) $$\begin{array}{cc} I_{rx} & =I_{ry}=I_{rt}\\ I_{gx} & =I_{gy}=I_{gt} \end{array}$$

Additionally, the inertia of the moments of the rotor and gimbal along the spin axis are re-assigned the variable names $I_{rs}$ and $I_{gs}$ to more clearly distinguish between spin (sub-subscript “s”) and transverse (sub-subscript “t”).

Taking Equations (7)–(9) into Equation (6) and rearranging the results, the motion of the equations of GW expressed by the case coordinates ($\phi_{x},\phi_{y}$) are yielded as follows:

(11) $$M_{c}\left(x\right)\ddot{x}+C_{c}\left(x\right)\dot{x}=Q_{c}\left(x\right)T_{c}+F_{c}\left(x,\dot{x}\right)+F_{\omega }\left(x,\dot{x},\omega_{b},{\dot{\omega }}_{b}\right)$$

where:

$$\begin{array}{cc} & x={\left[\begin{array}{cc} \phi_{x} & \phi_{y} \end{array}\right]}^{T}\;\;T_{c}={\left[\begin{array}{cc} T_{cx} & T_{cy} \end{array}\right]}^{T}\\ & M_{c}\left(x\right)=\left[\begin{array}{cc} I_{1}C_{\theta_{z}} & I_{1}S_{\theta_{z}}\\ -I_{rt}S_{\theta_{z}} & I_{rt}C_{\theta_{z}} \end{array}\right]\;C_{c}\left(x\right)=\left[\begin{array}{cc} c_{x}C_{\theta_{z}} & c_{x}S_{\theta_{z}}\\ -c_{y}S_{\theta_{z}} & c_{y}C_{\theta_{z}} \end{array}\right]\\ & Q_{c}\left(x\right)=\left[\begin{array}{cc} C_{\theta_{z}} & S_{\theta_{z}}\\ -S_{\theta_{z}}C_{\theta_{x}} & C_{\theta_{z}}C_{\theta_{x}} \end{array}\right]\;\;\;F_{c}\left(x,\dot{x}\right)=\left[\begin{array}{c} f_{c1}\left(x,\dot{x}\right)\\ f_{c2}\left(x,\dot{x}\right) \end{array}\right]\\ & F_{\omega }\left(x,\dot{x},\omega_{b},{\dot{\omega }}_{b}\right)=\left[\begin{array}{c} f_{\omega 1}\left(x,\dot{x},\omega_{b},{\dot{\omega }}_{b}\right)\\ f_{\omega 2}\left(x,\dot{x},\omega_{b},{\dot{\omega }}_{b}\right) \end{array}\right] \end{array}$$

$$\begin{array}{cc} & f_{c1}\left(x,\dot{x}\right)=-M_{c1}\cdot {\ddot{\theta }}_{z}-K_{c11}\cdot \phi_{x}-K_{c12}\cdot \phi_{y}-C_{c11}\cdot {\dot{\phi }}_{x}-C_{c12}\cdot {\dot{\phi }}_{y}-\frac{1}{2}I_{2}S_{2\theta_{x}}{\dot{\theta }}_{z}^{2}-I_{re}S_{2\theta_{y}}{\dot{\theta }}_{x}{\dot{\theta }}_{y}\\ & f_{c2}\left(x,\dot{x}\right)=-M_{c2}\cdot {\ddot{\theta }}_{z}-K_{c21}\cdot \phi_{x}-K_{c22}\cdot \phi_{y}-C_{c21}\cdot {\dot{\phi }}_{x}-C_{c22}\cdot {\dot{\phi }}_{y}-\frac{1}{2}I_{re}C_{\theta_{x}}^{2}S_{2\theta_{y}}{\dot{\theta }}_{z}^{2}+\frac{1}{2}I_{re}S_{2\theta_{y}}{\dot{\theta }}_{x}^{2}\\ & \begin{array}{cc} f_{\omega 1}\left(x,\dot{x},\omega_{b},{\dot{\omega }}_{b}\right) & =M_{1}\left(x,\dot{x}\right)\cdot {\dot{\omega }}_{bx}+M_{2}\left(x,\dot{x}\right)\cdot {\dot{\omega }}_{by}+M_{3}\left(x,\dot{x}\right)\cdot \omega_{bx}+M_{4}\left(x,\dot{x}\right)\cdot \omega_{by} \end{array}\\ & \begin{array}{cc} f_{\omega 2}\left(x,\dot{x},\omega_{b},{\dot{\omega }}_{b}\right) & =N_{1}\left(x,\dot{x}\right)\cdot {\dot{\omega }}_{bx}+N_{2}\left(x,\dot{x}\right)\cdot {\dot{\omega }}_{by}+N_{3}\left(x,\dot{x}\right)\cdot \omega_{bx}+N_{4}\left(x,\dot{x}\right)\cdot \omega_{by} \end{array} \end{array}$$

where:

$$\begin{array}{cc} & M_{c1}=I_{1}\cdot \left(\phi_{y}C_{\theta_{z}}-\phi_{x}S_{\theta_{z}}\right)+\frac{1}{2}I_{re}C_{\theta_{x}}S_{2\theta_{y}}\\ & M_{c2}=-I_{rt}\left(\phi_{x}C_{\theta_{z}}+\phi_{y}S_{\theta_{z}}-S_{\theta_{x}}\right)\\ & K_{c11}=-I_{1}{\dot{\theta }}_{z}^{2}C_{\theta_{z}}-c_{x}{\dot{\theta }}_{z}S_{\theta_{z}}+k_{x}C_{\theta_{z}}-I_{4}{\dot{\theta }}_{z}C_{\theta_{z}}\\ & K_{c12}=-I_{1}{\dot{\theta }}_{z}^{2}S_{\theta_{z}}+c_{x}{\dot{\theta }}_{z}C_{\theta_{z}}+k_{x}S_{\theta_{z}}-I_{4}{\dot{\theta }}_{z}S_{\theta_{z}}\\ & K_{c21}=I_{rt}{\dot{\theta }}_{z}^{2}S_{\theta_{z}}-c_{y}{\dot{\theta }}_{z}C_{\theta_{z}}-k_{y}S_{\theta_{z}}+I_{4}{\dot{\theta }}_{z}S_{\theta_{z}}\\ & K_{c22}=-I_{rt}{\dot{\theta }}_{z}^{2}C_{\theta_{z}}-c_{y}{\dot{\theta }}_{z}S_{\theta_{z}}+k_{y}C_{\theta_{z}}-I_{4}{\dot{\theta }}_{z}C_{\theta_{z}}\\ & C_{c11}=-2I_{1}{\dot{\theta }}_{z}S_{\theta_{z}}-I_{4}S_{\theta_{z}}\;C_{c12}=2I_{1}{\dot{\theta }}_{z}C_{\theta_{z}}+I_{4}C_{\theta_{z}}\\ & C_{c21}=-2I_{rt}{\dot{\theta }}_{z}C_{\theta_{z}}-I_{4}C_{\theta_{z}}\;C_{c22}=-2I_{rt}{\dot{\theta }}_{z}S_{\theta_{z}}-I_{4}S_{\theta_{z}}\\ & I_{4}=\left(I_{re}\cos\left(2\theta_{y}\right)-I_{rt}\right){\dot{\theta }}_{z}\cos\theta_{x} \end{array}$$

$M_{i}\left(x,\dot{x}\right),N_{i}\left(x,\dot{x}\right),i=1,\cdots ,4$ are equal to the corresponding expressions $B_{i},D_{i},i=1,\cdots ,4$; however, the variables $\theta_{j},{\dot{\theta }}_{j},j=x,y$ in $B_{i},D_{i}$ are substituted with Equations (7)–(9).

## 3. Modeling of Angular Rate Sensing with a Gyrowheel

### 3.1. Measurement Model at Arbitrary Operating Position Based on Real-Time Lyapunov Linearization

Suppose that a physical system can be generally expressed by the following nonlinear autonomous system equation:

(12) $$\dot{x}=f\left(x\right)+g\left(x\right)u$$

where $x={\left[x_{1},x_{2},\cdots ,x_{n}\right]}^{T}\in R^{n\times 1}$ is the state vector, $u={\left[u_{1},u_{2},\cdots ,u_{m}\right]}^{T}\in R^{m\times 1}$ is the control input vector and $f\left(x\right)\in R^{n\times 1}$ and $g\left(x\right)\in R^{n\times 1}$ are vector functions of states.

Assume $x_{d}\left(t\right)\in R^{n\times 1}$ is a given reference trajectory whose corresponding reference input is $u_{d}$, then we have:

(13) $${\dot{x}}_{d}=f\left(x_{d}\right)+g\left(x_{d}\right)u_{d}$$

Taking Lyapunov’s linearization [24,25] around the operating points $\left(x_{d},u_{d}\right)$, then it yields:

(14) $$\dot{x}={\dot{x}}_{d}+A\left(x_{d}\right)\left(x-x_{d}\right)+B\left(x_{d}\right)\left(u-u_{d}\right)$$

where $A\left(x_{d}\right)={\left.\frac{df}{dx}\right|}_{x=x_{d}},B\left(x_{d}\right)=g\left(x_{d}\right)$.

For the GW system, let $x_{d},{\dot{x}}_{d},{\ddot{x}}_{d},\omega_{bd},{\dot{\omega }}_{bd}$ be the operating points, which are given by:

(15) $$x_{d}^{T}=\left[\begin{array}{c} \phi_{xd}\\ \phi_{yd} \end{array}\right],{\dot{x}}_{d}^{T}=\left[\begin{array}{c} 0\\ 0 \end{array}\right],{\ddot{x}}_{d}^{T}=\left[\begin{array}{c} 0\\ 0 \end{array}\right],\omega_{bd}^{T}=\left[\begin{array}{c} 0\\ 0 \end{array}\right],{\dot{\omega }}_{bd}^{T}=\left[\begin{array}{c} 0\\ 0 \end{array}\right]$$

According to Lyapunov’s linearization theory expressed by Equations (13) and (14), the linearized dynamics equations of the GW are given by:

(16) $$\begin{array}{cc} & \frac{\partial \left(M_{c}\left(x\right)\ddot{x}\right)}{\partial \ddot{x}}{|}_{x=x_{d}}\left(\ddot{x}-{\ddot{x}}_{d}\right)+\frac{\partial \left(M_{c}\left(x\right)\ddot{x}\right)}{\partial x}{|}_{x=x_{d}}\left(x-x_{d}\right)\\ & \;+\frac{\partial \left(C_{c}\left(x\right)\dot{x}\right)}{\partial \dot{x}}{|}_{x=x_{d}}\left(\dot{x}-{\dot{x}}_{d}\right)=\left(\frac{\partial F_{c}}{\partial x}\right){|}_{\begin{array}{c} x=x_{d}\\ \dot{x}={\dot{x}}_{d} \end{array}}\left(x-x_{d}\right)\\ & \;+\left(\frac{\partial F_{c}}{\partial \dot{x}}\right){|}_{\begin{array}{c} x=x_{d}\\ \dot{x}={\dot{x}}_{d} \end{array}}\left(\dot{x}-{\dot{x}}_{d}\right)+\left(\frac{\partial F_{\omega }}{\partial \omega_{b}}\right){|}_{\begin{array}{c} x=x_{d}\\ \dot{x}={\dot{x}}_{d} \end{array}}\left(\omega_{b}-\omega_{bd}\right)\\ & \;+\left(\frac{\partial F_{\omega }}{\partial {\dot{\omega }}_{b}}\right){|}_{x=x_{d},\dot{x}={\dot{x}}_{d}}\left({\dot{\omega }}_{b}-{\dot{\omega }}_{bd}\right)\\ & \;+Q_{c}\left(x_{d}\right)\cdot \left(T_{c}-T_{cd}\right)+F_{h.o.t}\left(x,\dot{x},\omega_{bd},{\dot{\omega }}_{bd}\right) \end{array}$$

where:

$$\begin{array}{c} \frac{\partial \left(M_{c}\left(x\right)\ddot{x}\right)}{\partial \ddot{x}}{|}_{x=x_{d}}={\left[\begin{array}{cc} I_{1}C_{\theta_{z}} & I_{1}S_{\theta_{z}}\\ -I_{rt}S_{\theta_{z}} & I_{rt}C_{\theta_{z}} \end{array}\right]}_{x=x_{d}}\;Q_{c}\left(x_{d}\right)={\left[\begin{array}{cc} C_{\theta_{z}} & S_{\theta_{z}}\\ -S_{\theta_{z}}C_{\theta_{x}} & C_{\theta_{z}}C_{\theta_{x}} \end{array}\right]}_{x=x_{d}} \end{array}$$

$$\begin{array}{c} \frac{\partial \left(M_{c}\left(x\right)\ddot{x}\right)}{\partial x}{|}_{x=x_{d}}={\left[\begin{array}{cc} -I_{re}S_{2\theta_{y}}S_{\theta_{z}}\left(C_{\theta_{z}}{\ddot{\phi }}_{x}+S_{\theta_{z}}{\ddot{\phi }}_{y}\right) & I_{re}S_{2\theta_{y}}C_{\theta_{z}}\left(C_{\theta_{z}}{\ddot{\phi }}_{x}+S_{\theta_{z}}{\ddot{\phi }}_{y}\right)\\ 0 & 0 \end{array}\right]}_{x=x_{d}} \end{array}$$

$$\begin{array}{cc} & \left(\frac{\partial F_{c}}{\partial x}\right){|}_{x=x_{d},\dot{x}={\dot{x}}_{d}}={\left[\begin{array}{cc} \frac{\partial f_{c1}}{\partial \phi_{x}} & \frac{\partial f_{c1}}{\partial \phi_{y}}\\ \frac{\partial f_{c2}}{\partial \phi_{x}} & \frac{\partial f_{c2}}{\partial \phi_{y}} \end{array}\right]}_{x=x_{d},\dot{x}={\dot{x}}_{d}}\;\left(\frac{\partial F_{c}}{\partial \dot{x}}\right){|}_{x=x_{d},\dot{x}={\dot{x}}_{d}}={\left[\begin{array}{cc} \frac{\partial f_{c1}}{\partial {\dot{\phi }}_{x}} & \frac{\partial f_{c1}}{\partial {\dot{\phi }}_{y}}\\ \frac{\partial f_{c2}}{\partial {\dot{\phi }}_{x}} & \frac{\partial f_{c2}}{\partial {\dot{\phi }}_{y}} \end{array}\right]}_{x=x_{d},\dot{x}={\dot{x}}_{d}}\\ & \left(\frac{\partial F_{\omega }}{\partial \omega_{b}}\right){|}_{x=x_{d},\dot{x}={\dot{x}}_{d}}={\left[\begin{array}{cc} \frac{\partial f_{c1}}{\partial \omega_{bx}} & \frac{\partial f_{c1}}{\partial \omega_{by}}\\ \frac{\partial f_{c2}}{\partial \omega_{bx}} & \frac{\partial f_{c2}}{\partial \omega_{by}} \end{array}\right]}_{x=x_{d},\dot{x}={\dot{x}}_{d}}\left(\frac{\partial F_{\omega }}{\partial {\dot{\omega }}_{b}}\right){|}_{x=x_{d},\dot{x}={\dot{x}}_{d}}={\left[\begin{array}{cc} \frac{\partial f_{c1}}{\partial {\dot{\omega }}_{bx}} & \frac{\partial f_{c1}}{\partial {\dot{\omega }}_{by}}\\ \frac{\partial f_{c2}}{\partial {\dot{\omega }}_{bx}} & \frac{\partial f_{c2}}{\partial {\dot{\omega }}_{by}} \end{array}\right]}_{x=x_{d},\dot{x}={\dot{x}}_{d}} \end{array}$$

$F_{h.o.t}\left(x,\dot{x},\omega_{bd},{\dot{\omega }}_{bd}\right)$ are the high order terms and will be ignored in the following. For brevity, the elements of the Jacobian matrices of the vectors $F_{c},F_{\omega }$ over the vectors $x,\dot{x},\omega_{b},{\dot{\omega }}_{b}$, such as $\frac{\partial f_{ci}}{\partial \phi_{j}},\frac{\partial f_{ci}}{\partial {\dot{\phi }}_{j}},\frac{\partial f_{ci}}{\partial \omega_{bj}},\frac{\partial f_{ci}}{\partial {\dot{\omega }}_{bj}},i=1,2,j=x,y$, are also ignored here.

For the equilibrium points of the control input torque vector $T_{cd}$, the following constraint condition holds:

(17) $$\begin{array}{cc} M_{c}\left(x_{d}\right){\ddot{x}}_{d} & +C_{c}\left(x_{d}\right){\dot{x}}_{d}=Q_{c}\left(x_{d}\right)T_{cd}+F_{c}\left(x_{d},{\dot{x}}_{d}\right)+F_{\omega }\left(x_{d},{\dot{x}}_{d},\omega_{b},{\dot{\omega }}_{b}\right) \end{array}$$

Substituting Equation (15) into Equation (17), we have the constraint relationship between the operating state $x_{d}$ and the nominal control input torque $T_{cd}$ as follows:

(18) $$T_{cd}=-Q_{c}^{-1}\left(x_{d}\right)\cdot \left(F_{c}\left(x_{d},0\right)+F_{\omega }\left(x_{d},0,0,0\right)\right)$$

Finally, combining Equation (16) with Equation (15) and rearranging the results, the yielded GW Lyapunov linearized equations are given by:

(19) $$\begin{array}{cc} B_{c}^{\prime }\left(x_{d}\right)\left[\begin{array}{c} {\dot{\omega }}_{bx}\\ {\dot{\omega }}_{by} \end{array}\right] & +D_{c}^{\prime }\left(x_{d}\right)\left[\begin{array}{c} \omega_{bx}\\ \omega_{by} \end{array}\right]=M_{c}^{\prime }\left(x_{d}\right)\left[\begin{array}{c} {\ddot{\phi }}_{x}\\ {\ddot{\phi }}_{y} \end{array}\right]+C_{c}^{\prime }\left(x_{d}\right)\left[\begin{array}{c} {\dot{\phi }}_{x}\\ {\dot{\phi }}_{y} \end{array}\right]\\ & +K_{c}^{\prime }\left(x_{d}\right)\left[\begin{array}{c} \phi_{x}-\phi_{xd}\\ \phi_{y}-\phi_{yd} \end{array}\right]-Q_{c}\left(x_{d}\right)\left[\begin{array}{c} T_{cx}-T_{cxd}\\ T_{cy}-T_{cyd} \end{array}\right] \end{array}$$

where:

$$\begin{array}{cc} M_{c}^{\prime }\left(x_{d}\right) & ={\left[\begin{array}{cc} I_{1}C_{\theta_{z}}+\chi_{1}\left(x_{d}\right) & I_{1}S_{\theta_{z}}+\chi_{2}\left(x_{d}\right)\\ -I_{rt}S_{\theta_{z}} & I_{rt}C_{\theta_{z}} \end{array}\right]}_{x=x_{d}}\\ C_{c}^{\prime }\left(x_{d}\right) & ={\left[\begin{array}{cc} c_{x}C_{\theta_{z}}-\frac{\partial f_{c1}\left(x,\dot{x}\right)}{\partial {\dot{\phi }}_{x}} & c_{x}S_{\theta_{z}}-\frac{\partial f_{c1}\left(x,\dot{x}\right)}{\partial {\dot{\phi }}_{y}}\\ -c_{y}S_{\theta_{z}}-\frac{\partial f_{c2}\left(x,\dot{x}\right)}{\partial {\dot{\phi }}_{x}} & c_{y}C_{\theta_{z}}-\frac{\partial f_{c2}\left(x,\dot{x}\right)}{\partial {\dot{\phi }}_{y}} \end{array}\right]}_{x=x_{d},\dot{x}={\dot{x}}_{d}}\\ K_{c}^{\prime }\left(x_{d}\right) & ={\left[\begin{array}{cc} \frac{\partial f_{c1}\left(x,\dot{x}\right)}{\partial \phi_{x}} & \frac{\partial f_{c1}\left(x,\dot{x}\right)}{\partial \phi_{y}}\\ \frac{\partial f_{c2}\left(x,\dot{x}\right)}{\partial \phi_{x}} & \frac{\partial f_{c2}\left(x,\dot{x}\right)}{\partial \phi_{y}} \end{array}\right]}_{x=x_{d},\dot{x}={\dot{x}}_{d}}\\ B_{c}^{\prime }\left(x_{d}\right) & ={\left[\begin{array}{cc} M_{1}\left(x,\dot{x}\right) & M_{2}\left(x,\dot{x}\right)\\ N_{1}\left(x,\dot{x}\right) & N_{2}\left(x,\dot{x}\right) \end{array}\right]}_{x=x_{d},\dot{x}={\dot{x}}_{d}}D_{c}^{\prime }\left(x_{d}\right)={\left[\begin{array}{cc} M_{3}\left(x,\dot{x}\right) & M_{4}\left(x,\dot{x}\right)\\ N_{3}\left(x,\dot{x}\right) & N_{4}\left(x,\dot{x}\right) \end{array}\right]}_{x=x_{d},\dot{x}={\dot{x}}_{d}}\\ \chi_{1}\left(x_{d}\right) & =I_{re}S_{2\theta_{y}}\cdot \left(-\frac{1}{2}S_{2\theta_{z}}\left(\phi_{x}-\phi_{xd}\right)-C_{\theta_{z}}^{2}\left(\phi_{y}-\phi_{yd}\right)\right)\\ \chi_{2}\left(x_{d}\right) & =I_{re}S_{2\theta_{y}}\cdot \left(-S_{2\theta_{z}}^{2}\left(\phi_{x}-\phi_{xd}\right)+\frac{1}{2}S_{2\theta_{z}}^{2}\left(\phi_{y}-\phi_{yd}\right)\right) \end{array}$$

When the operating points $x_{d}$ are given by the tilt control commands, which are supplied by ACS, the real-time Lyapunov’s linearization of the GW measurement equations can be realized in theory.

### 3.2. Measurement Model within Small Tilt Angles Based on Complex Quantity Transform

Specially, when the operating points $x_{d}$ are further considered to be set as zero tilt angles, that is, $x_{d}^{T}=\left[\begin{array}{c} \phi_{xd}\\ \phi_{yd} \end{array}\right]=\left[\begin{array}{c} 0\\ 0 \end{array}\right]$, we have:

(20) $$\begin{array}{cc} \left[\begin{array}{cc} I_{t}C_{\theta_{z}} & I_{t}S_{\theta_{z}}\\ -I_{rt}S_{\theta_{z}} & I_{rt}C_{\theta_{z}} \end{array}\right]\left[\begin{array}{c} {\ddot{\phi }}_{x}\\ {\ddot{\phi }}_{y} \end{array}\right] & +\left[\begin{array}{cc} \phi_{d11} & \phi_{d12}\\ \phi_{d21} & \phi_{d22} \end{array}\right]\left[\begin{array}{c} {\dot{\phi }}_{x}\\ {\dot{\phi }}_{y} \end{array}\right]-\left[\begin{array}{cc} \phi_{11} & \phi_{12}\\ \phi_{21} & \phi_{22} \end{array}\right]\left[\begin{array}{c} \phi_{x}\\ \phi_{y} \end{array}\right]=\left[\begin{array}{cc} C_{\theta_{z}} & S_{\theta_{z}}\\ -S_{\theta_{z}} & C_{\theta_{z}} \end{array}\right]\left[\begin{array}{c} T_{cx}\\ T_{cy} \end{array}\right]\\ & +\left[\begin{array}{cc} -I_{t}C_{\theta_{z}} & -I_{t}S_{\theta_{z}}\\ I_{rt}S_{\theta_{z}} & -I_{rt}C_{\theta_{z}} \end{array}\right]\left[\begin{array}{c} {\dot{\omega }}_{bx}\\ {\dot{\omega }}_{by} \end{array}\right]+\left[\begin{array}{cc} I_{s}{\dot{\theta }}_{z}S_{\theta_{z}} & -I_{s}{\dot{\theta }}_{z}C_{\theta_{z}}\\ I_{rs}{\dot{\theta }}_{z}C_{\theta_{z}} & I_{rs}{\dot{\theta }}_{z}S_{\theta_{z}} \end{array}\right]\left[\begin{array}{c} \omega_{bx}\\ \omega_{by} \end{array}\right] \end{array}$$

where:

$$\begin{array}{cc} I_{t} & =I_{rt}+I_{gt}\;\;I_{s}=I_{rs}+I_{gs}\\ \phi_{d11} & =c_{x}C_{\theta_{z}}-\left(I_{rs}+2I_{gt}\right){\dot{\theta }}_{z}S_{\theta_{z}}\;\;\phi_{d12}=c_{x}S_{\theta_{z}}+\left(I_{rs}+2I_{gt}\right){\dot{\theta }}_{z}C_{\theta_{z}}\\ \phi_{d21} & =-c_{y}S_{\theta_{z}}-I_{rs}{\dot{\theta }}_{z}C_{\theta_{z}}\;\;\;\;\phi_{d22}=c_{y}C_{\theta_{z}}-I_{rs}{\dot{\theta }}_{z}S_{\theta_{z}}\\ \phi_{11} & =\left(I_{gt}+I_{rs}\right){\ddot{\theta }}_{z}S_{\theta_{z}}+c_{x}{\dot{\theta }}_{z}S_{\theta_{z}}-k_{x}C_{\theta_{z}}+\left(2I_{gt}-I_{gs}\right){\dot{\theta }}_{z}^{2}C_{\theta_{z}}\\ \phi_{12} & =-\left(I_{rs}+I_{gt}\right){\ddot{\theta }}_{z}C_{\theta_{z}}-c_{x}{\dot{\theta }}_{z}C_{\theta_{z}}-k_{x}S_{\theta_{z}}+\left(2I_{gt}-I_{gs}\right){\dot{\theta }}_{z}^{2}S_{\theta_{z}}\\ \phi_{21} & =I_{rt}{\ddot{\theta }}_{z}C_{\theta_{z}}+c_{y}{\dot{\theta }}_{z}C_{\theta_{z}}+k_{y}S_{\theta_{z}}\;\phi_{22}=I_{rt}{\ddot{\theta }}_{z}S_{\theta_{z}}+c_{y}{\dot{\theta }}_{z}S_{\theta_{z}}-k_{y}C_{\theta_{z}} \end{array}$$

Since the two-axis tilt angles of the rotor $\left(\phi_{x},\phi_{y}\right)$, the two-axis tilt control torques $\left(T_{cx},T_{cy}\right)$ and the defined two-axis spacecraft angular rates $\left(\omega_{bx},\omega_{by}\right)$ are perpendicular to each other, respectively, to represent the equation set Equation (20) in the form of one single complex equation [26], the following complex quantities are defined by:

(21) $$\begin{array}{c} \phi =\phi_{x}+j\phi_{y}\;T_{c}=T_{cx}+jT_{cy}\;\omega =\omega_{bx}+j\omega_{by} \end{array}$$

Then, the case-fixed tilt angles, control torques and spacecraft angular rates are expressed, in terms of the newly-defined complex variables and their complex conjugates, as follows:

(22) $$\left\{\begin{array}{c} \phi_{x}=\frac{1}{2}\left(\bar{\phi }+\phi \right)\\ \phi_{y}=\frac{j}{2}\left(\bar{\phi }-\phi \right) \end{array}\right.\;\left\{\begin{array}{c} T_{cx}=\frac{1}{2}\left({\bar{T}}_{c}+T_{c}\right)\\ T_{cy}=\frac{j}{2}\left({\bar{T}}_{c}-T_{c}\right) \end{array}\right.\;\left\{\begin{array}{c} \omega_{bx}=\frac{1}{2}\left({\bar{\omega }}_{b}+\omega_{b}\right)\\ \omega_{by}=\frac{j}{2}\left({\bar{\omega }}_{b}-\omega_{b}\right) \end{array}\right.$$

The first row of Equation (20) is added to $j=\sqrt{-1}$ times the second row of Equation (20), then substituting Equation (22) into the result and considering the relationship $\prime \prime e^{j\theta_{z}}=\cos\theta_{z}+j\sin\theta_{z}^{\prime \prime }$, the following single differential equation with complex coefficients is yielded:

(23) $$\begin{array}{cc} & \left(I_{rt}+\frac{1}{2}I_{gt}\right)\ddot{\phi }e^{-j\theta_{z}}+\frac{1}{2}I_{gt}\ddot{\bar{\phi }}e^{j\theta_{z}}+\left(c_{g}-j\left(I_{rs}+I_{gt}\right){\dot{\theta }}_{z}\right)\dot{\phi }e^{-j\theta_{z}}+jI_{gt}{\dot{\theta }}_{z}\dot{\bar{\phi }}e^{j\theta_{z}}\\ & +\left[\frac{K_{x}+K_{y}}{2}-\left(I_{gt}-\frac{1}{2}I_{gs}\right){\dot{\theta }}_{z}^{2}-j\left(c_{g}{\dot{\theta }}_{z}+\frac{I_{gt}+I_{rt}+I_{rs}}{2}{\ddot{\theta }}_{z}\right)\right]\phi e^{-j\theta_{z}}\\ & +\left[\frac{K_{x}-K_{y}}{2}-\left(I_{gt}-\frac{1}{2}I_{gs}\right){\dot{\theta }}_{z}^{2}-j\frac{I_{rt}-I_{gt}-I_{rs}}{2}{\ddot{\theta }}_{z}\right]\bar{\phi }e^{j\theta_{z}}\\ & =-\left(I_{rt}+\frac{1}{2}I_{gt}\right){\dot{\omega }}_{b}e^{-j\theta_{z}}-\frac{1}{2}I_{gt}{\dot{\bar{\omega }}}_{b}e^{j\theta_{z}}+j\left(I_{rs}+\frac{1}{2}I_{gs}\right){\dot{\theta }}_{z}\omega_{b}e^{-j\theta_{z}}-j\frac{1}{2}I_{gs}{\dot{\theta }}_{z}{\bar{\omega }}_{b}e^{j\theta_{z}}+T_{c}e^{-j\theta_{z}} \end{array}$$

Rearranging Equation (23), we have:

(24) $$\begin{array}{c} e^{-j\theta_{z}}\left\{\begin{array}{cc} & \ddot{\phi }+\left(\frac{2c_{g}}{2I_{rt}+I_{gt}}-j\left(\frac{2I_{rs}}{2I_{rt}+I_{gt}}+\frac{2I_{gt}}{2I_{rt}+I_{gt}}\right){\dot{\theta }}_{z}\right)\dot{\phi }\\ & +\left[\frac{K_{x}+K_{y}-2\left(I_{gt}-\frac{1}{2}I_{gs}\right){\dot{\theta }}_{z}^{2}}{2I_{rt}+I_{gt}}-j\left(\frac{2c_{g}}{2I_{rt}+I_{gt}}{\dot{\theta }}_{z}+\frac{I_{gt}+I_{rt}+I_{rs}}{2I_{rt}+I_{gt}}{\ddot{\theta }}_{z}\right)\right]\phi \\ & +{\dot{\omega }}_{b}-j\left(\frac{2I_{rs}}{2I_{rt}+I_{gt}}+\frac{I_{gs}}{2I_{rt}+I_{gt}}\right){\dot{\theta }}_{z}\omega_{b}\\ & +e^{j2\theta_{z}}\left\{\begin{array}{cc} & \frac{I_{gt}}{2I_{rt}+I_{gt}}\ddot{\bar{\phi }}+j\frac{2I_{gt}}{2I_{rt}+I_{gt}}{\dot{\theta }}_{z}\dot{\bar{\phi }}\\ & +\left[\frac{K_{x}-K_{y}-2\left(I_{gt}-\frac{1}{2}I_{gs}\right){\dot{\theta }}_{z}^{2}}{2I_{rt}+I_{gt}}-j\frac{I_{rt}-I_{gt}-I_{rs}}{2I_{rt}+I_{gt}}{\ddot{\theta }}_{z}\right]\bar{\phi }\\ & +\frac{I_{gt}}{2I_{rt}+I_{gt}}{\dot{\bar{\omega }}}_{b}+j\frac{I_{gs}}{2I_{rt}+I_{gt}}{\dot{\theta }}_{z}{\bar{\omega }}_{b} \end{array}\right\} \end{array}\right\}=e^{-j\theta_{z}}\cdot \frac{T_{c}}{I_{rt}+\frac{1}{2}I_{gt}} \end{array}$$

For conciseness, the following quantities are defined by:

$$\begin{array}{cc} c_{g}^{\prime } & =\frac{c_{g}}{2I_{rt}+I_{gt}}\;\omega_{n}=\frac{2I_{rs}}{2I_{rt}+I_{gt}}{\dot{\theta }}_{z}\;\gamma =\frac{I_{gt}}{2I_{rt}+I_{gt}}\;\gamma_{2}=\frac{I_{gs}}{2I_{rt}+I_{gt}}\\ \alpha & =\frac{K_{x}+K_{y}-2\left(I_{gt}-\frac{1}{2}I_{gs}\right){\dot{\theta }}_{z}^{2}}{2I_{rt}+I_{gt}}\;\beta =\frac{K_{x}-K_{y}-2\left(I_{gt}-\frac{1}{2}I_{gs}\right){\dot{\theta }}_{z}^{2}}{2I_{rt}+I_{gt}}\\ I_{rt}^{\prime } & =I_{rt}+\frac{1}{2}I_{gt}\;J_{p}=\frac{I_{gt}+I_{rt}+I_{rs}}{2I_{rt}+I_{gt}}\;J_{m}=\frac{I_{rt}-I_{gt}-I_{rs}}{2I_{rt}+I_{gt}} \end{array}$$

and taking the newly-defined quantities into Equation (24) yields:

(25) $$\begin{array}{c} e^{-j\theta_{z}}\left\{\begin{array}{cc} & \ddot{\phi }+\left(2c_{g}^{\prime }-j\left(\omega_{n}+2\gamma {\dot{\theta }}_{z}\right)\right)\dot{\phi }\\ & +\left[\alpha -j\left(2C_{g}^{\prime }{\dot{\theta }}_{z}+J_{p}{\ddot{\theta }}_{z}\right)\right]\phi +{\dot{\omega }}_{b}-j\left(\omega_{n}+\gamma_{2}{\dot{\theta }}_{z}\right)\omega_{b}\\ & +e^{j2\theta_{z}}\left\{\gamma \ddot{\bar{\phi }}+j2\gamma {\dot{\theta }}_{z}\dot{\bar{\phi }}+\left(\beta -jJ_{m}{\ddot{\theta }}_{z}\right)\bar{\phi }+\gamma {\dot{\bar{\omega }}}_{b}+j\gamma_{2}{\dot{\theta }}_{z}{\bar{\omega }}_{b}\right\} \end{array}\right\}=\frac{T_{c}}{I_{rt}^{\prime }}\cdot e^{-j\theta_{z}} \end{array}$$

Eliminating the factor $e^{-j\theta_{z}}$ in Equation (25), we obtain the more concise form of the GW linearization equations at zero tilt angles given by:

(26) $$\begin{array}{cc} & \ddot{\phi }+\left(2c_{g}^{\prime }-j\left(\omega_{n}+2\gamma {\dot{\theta }}_{z}\right)\right)\dot{\phi }+\left[\alpha -j\left(2c_{g}^{\prime }{\dot{\theta }}_{z}+J_{p}{\ddot{\theta }}_{z}\right)\right]\phi +{\dot{\omega }}_{b}-j\left(\omega_{n}+\gamma_{2}{\dot{\theta }}_{z}\right)\omega_{b}\\ & +e^{j2\theta_{z}}\left[\gamma \ddot{\bar{\phi }}+j2\gamma {\dot{\theta }}_{z}\dot{\bar{\phi }}+\left(\beta -jJ_{m}{\ddot{\theta }}_{z}\right)\bar{\phi }+\gamma {\dot{\bar{\omega }}}_{b}+j\gamma_{2}{\dot{\theta }}_{z}{\bar{\omega }}_{b}\right]=\frac{T_{c}}{I_{rt}^{\prime }} \end{array}$$

Finally, substituting Equation (21) into Equation (26), substituting $e^{2\theta_{z}}$ with $C_{2\theta_{z}}+jS_{2\theta_{z}}$ and restoring the results expressed by the complex quantity into real-value equations, we obtain the GW linearization equations at zero tilt angles represented in the real-value form:

(27) $$\begin{array}{cc} & \left[\begin{array}{cc} 1+\gamma C_{2\theta_{z}} & \gamma S_{2\theta_{z}}\\ \gamma S_{2\theta_{z}} & 1-\gamma C_{2\theta_{z}} \end{array}\right]\left[\begin{array}{c} {\ddot{\phi }}_{x}\\ {\ddot{\phi }}_{y} \end{array}\right]+\left[\begin{array}{cc} 2c_{g}^{\prime }-2\gamma {\dot{\theta }}_{z}S_{2\theta_{z}} & \left(\omega_{n}+2\gamma {\dot{\theta }}_{z}\right)+2\gamma {\dot{\theta }}_{z}C_{2\theta_{z}}\\ -\left(\omega_{n}+2\gamma {\dot{\theta }}_{z}\right)+2\gamma {\dot{\theta }}_{z}C_{2\theta_{z}} & 2c_{g}^{\prime }+2\gamma {\dot{\theta }}_{z}S_{2\theta_{z}} \end{array}\right]\left[\begin{array}{c} {\dot{\phi }}_{x}\\ {\dot{\phi }}_{y} \end{array}\right]\\ & +\left[\begin{array}{cc} \alpha +\beta C_{2\theta_{z}} & 2c_{g}^{\prime }{\dot{\theta }}_{z}+\beta S_{2\theta_{z}}+\left(J_{p}-J_{m}C_{2\theta_{z}}+J_{m}S_{2\theta_{z}}\right){\ddot{\theta }}_{z}\\ -2c_{g}^{\prime }{\dot{\theta }}_{z}+\beta S_{2\theta_{z}}-\left(J_{p}+J_{m}C_{2\theta_{z}}-J_{m}S_{2\theta_{z}}\right){\ddot{\theta }}_{z} & \alpha -\beta C_{2\theta_{z}} \end{array}\right]\left[\begin{array}{c} \phi_{x}\\ \phi_{y} \end{array}\right]\\ & +\left[\begin{array}{cc} 1+\gamma C_{2\theta_{z}} & \gamma S_{2\theta_{z}}\\ \gamma S_{2\theta_{z}} & 1-\gamma C_{2\theta_{z}} \end{array}\right]\left[\begin{array}{c} {\dot{\omega }}_{bx}\\ {\dot{\omega }}_{by} \end{array}\right]+\left[\begin{array}{cc} -\gamma_{2}{\dot{\theta }}_{z}S_{2\theta_{z}} & \left(\omega_{n}+\gamma_{2}{\dot{\theta }}_{z}\right)+\gamma_{2}{\dot{\theta }}_{z}C_{2\theta_{z}}\\ -\left(\omega_{n}+\gamma_{2}{\dot{\theta }}_{z}\right)+\gamma_{2}{\dot{\theta }}_{z}C_{2\theta_{z}} & \gamma_{2}{\dot{\theta }}_{z}S_{2\theta_{z}} \end{array}\right]\left[\begin{array}{c} \omega_{bx}\\ \omega_{by} \end{array}\right]=\frac{1}{I_{rt}^{{}^{\prime }}}\left[\begin{array}{c} T_{cx}\\ T_{cy} \end{array}\right] \end{array}$$

From the left side of Equation (27), each element of the coefficient matrices contains the twice periodic components about the motor spin speed ${\dot{\theta }}_{z}$. Since the periodic terms have no effects on the measurement accuracy of the spacecraft angular rates ($\omega_{bx},\omega_{by}$) and ignoring the periodic terms will save the unnecessary sensor for measuring the motor rotation angle $\theta_{z}$, therefore, the periodic terms are ignored with the model simplifying to the following form:

(28) $$\begin{array}{cc} \left[\begin{array}{c} {\ddot{\phi }}_{x}\\ {\ddot{\phi }}_{y} \end{array}\right]+ & \left[\begin{array}{cc} 2c_{g}^{\prime } & \left(\omega_{n}+2\gamma {\dot{\theta }}_{z}\right)\\ -\left(\omega_{n}+2\gamma {\dot{\theta }}_{z}\right) & 2c_{g}^{\prime } \end{array}\right]\left[\begin{array}{c} {\dot{\phi }}_{x}\\ {\dot{\phi }}_{y} \end{array}\right]+\left[\begin{array}{cc} \alpha & \left(2c_{g}^{\prime }{\dot{\theta }}_{z}+J_{p}{\ddot{\theta }}_{z}\right)\\ -\left(2c_{g}^{\prime }{\dot{\theta }}_{z}+J_{p}{\ddot{\theta }}_{z}\right) & \alpha \end{array}\right]\left[\begin{array}{c} \phi_{x}\\ \phi_{y} \end{array}\right]\\ & +\left[\begin{array}{c} {\dot{\omega }}_{bx}\\ {\dot{\omega }}_{by} \end{array}\right]+\left[\begin{array}{cc} 0 & \left(\omega_{n}+\gamma_{2}{\dot{\theta }}_{z}\right)\\ -\left(\omega_{n}+\gamma_{2}{\dot{\theta }}_{z}\right) & 0 \end{array}\right]\left[\begin{array}{c} \omega_{bx}\\ \omega_{by} \end{array}\right]=\frac{1}{I_{rt}^{\prime }}\left[\begin{array}{c} T_{cx}\\ T_{cy} \end{array}\right] \end{array}$$

## 4. Analysis of the Measurement Schemes of Spacecraft Angular Rates with the GW

In order to analyze the proposed measurement equations, the following two definitions are given:

**Definition 1.**  *If the tilt angle of the GW rotor works at a certain tilt position and remains unchanged as the spacecraft angular rates are measured with the GW, we call this situation* “StaticMeasurement”.

**Definition 2.**  *In contrast to Definition 1, when the tilt angular velocity of the GW rotor is nonzero as the spacecraft angular rates are measured with the GW, we call this situation* “DynamicMeasurement”.

From the derived measurement Equations (20) and (28), three pieces of valuable information are summarized as follows:

(1) Compared to Equation (20), the forms of linearization Equation (28) at zero tilt angles are obviously more concise. More importantly, after ignoring the twice periodic components in Equation (27), there exist no terms about motor rotation angle $\theta_{z}$ in Equation (28), which means that it is more convenient for the spacecraft angular rate sensing, since the special sensor for measuring the motor rotation angle in real time is no longer needed in this kind of situation.
(2) Equation (28) is obtained by substituting the operating points at zero tilt angles into Equation (20), which are suitable for the arbitrary operating state of the rotor. Therefore, combining the analysis (1), in the smaller tilt range of the rotor, Equation (28) is utilized to realize the spacecraft angular rate sensing with GW. However, when the tilt angles of the rotor become larger, the measurement accuracy with Equation (28) cannot meet the indicator requirement without any error compensation. The applications of the real-time linearization measurement Equation (20) and small tilt measurement Equation (28) are analyzed in the following figure.

**Figure 3:**
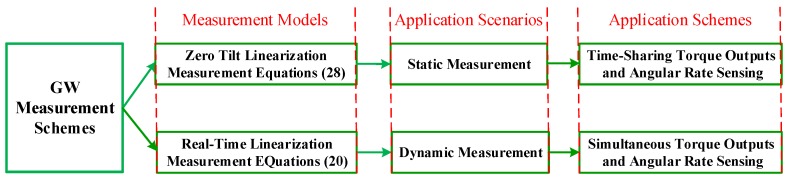
Measurement schemes of spacecraft angular rates with GW in different working modes.

In Figure 3, considering GW in the application scenario of static measurement, when the rotor works at large tilt angles, the measurement errors due to the linearization at zero tilt angles are significantly correlated to the tilt angles of the rotor, which can be modeled and compensated in the measurement Equation (28) by the methods of polynomial fitting, B-spline functions or table lookups based on the calibration data. Since the rotor needs to keep still for realizing the spacecraft angular rate sensing in this situation, the radial control torques cannot be supplied by the GW, which means that with Equation (28) as the measurement equations of the GW, it has two kinds of working modes: the radial torque outputs mode and the spacecraft angular sensing mode, and these two modes cannot be realized at the same time, we can name the two working modes as ′′time-$sharing\;multiplexing^{\prime \prime }$. As previously mentioned, the greatest advantages of this scheme are that there is no need to set the rotary transformer for measuring the motor rotation angle $\theta_{z}$, and the regular errors like the linearization at zero tilt angles can be more easily calibrated by ground experiments. However, this scheme has an obvious drawback that GW cannot realize the radial torque outputs and angular rate sense simultaneously, so that the application of GW is limited in the area of spacecraft.

Whereas in the state of dynamic measurement, since the measurement errors are not only relevant to the tilt angles of the rotor, but also to the tilt angular velocities and accelerations, it is difficult for Equation (28) to compensate the linearization errors by polynomial fitting or B-spline functions due to the huge workload. Therefore, in the case of dynamic measurement, the real-time Lyapunov’s linearization measurement Equation (20) need to be applied to sense the spacecraft angular rates, which can avoid the errors of the small tilt linearization Equation (28) at larger tilt angles. Most important of all, gyroscopic moments can be generated at the same time because of the existence of the angular momentum and the tilt angular velocity of the rotor in dynamic measurement. However, the prices are that a rotary transformer must be assembled in the GW system to measure the motor rotation angle $\theta_{z}$, and the real-time disturbance estimation methods like [27,28] should be further developed to compensate regular mechanical errors for Equation (20), which will not be discussed in detail in this paper.

(3) Since the effects of the motor spin acceleration are considered in Equation (28), while the motor spin speed is being changed to control the spacecraft attitude along the spin axis, the angular rate sensing can be more accurately realized by Equation (28) in small tilt ranges.

## 5. Simulations

### 5.1. Simulation Platform

**Figure 4:**
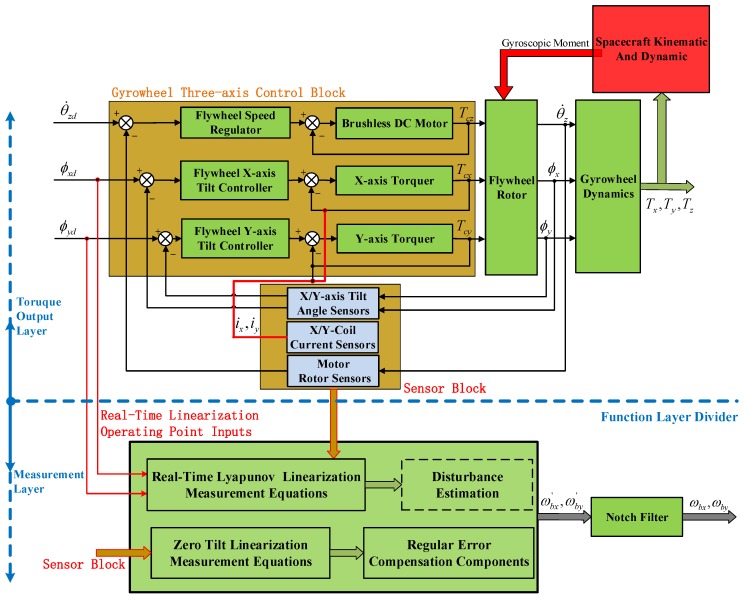
Simulation platform of spacecraft angular rate sensing based on the gyrowheel system.

To demonstrate the effectiveness of the proposed angular rate measurement approach, a simulation platform as in Figure 4 is built. The simulation platform is divided into two layers: the torque output layer and the measurement layer. The former contains the motor control loop, the *X*-axis and the *Y*-axis tilt control loops. The variables ${\dot{\theta }}_{zd},\phi_{xd},\phi_{yd}$ are the command inputs of the GW control loops. The variables ${\dot{\theta }}_{z},\phi_{x},\phi_{y}$ are the corresponding measurable outputs of the above three control loops, respectively. The variables $T_{cz},T_{cx},T_{cy}$ are control torques of the above three control loops, where $T_{cz}$ is used to control the motor rotation speed, and the torques $T_{cx},T_{cy}$ make the rotor tilt along radial directions. The variables $T_{z},T_{x},T_{y}$ acting on the spacecraft block are the three-axis control torques generated by the GW system. The sensor block includes the *X*-axis, *Y*-axis tilt angle sensors, the currents of torquer coils, motor rotation speed and angle sensors, which can directly measure the variables $\phi_{x},\phi_{y},T_{cx},T_{cy},\theta_{z},{\dot{\theta }}_{z}$, respectively.

The measurement layer is designed to realize the angular rate sense of the spacecraft with the proposed measurement methods, which contains the real-time Lyapunov linearization Equation (20) and the small tilt linearization Equation (28). The tilt command inputs $\left(\phi_{xd},\phi_{yd}\right)$ as the desired operating points are utilized to realize the real-time Lyapunov linearization measurement. The outputs of the sensor block are input to both measurement equations. As previously mentioned, the disturbance estimation (refer to [27,28]) should be studied further for Equation (20) in engineering. The regular error compensation is investigated for Equation (28) for compensating the errors due to zero tilt angle linearization.

The spacecraft angular rates $\omega_{bx}^{\prime },\omega_{by}^{\prime }$ can be obtained from the proposed measurement equations; however, unlike the zero position linearization Equation (28), the twice periodic components are hardly separated and ignored from the real-time Lyapunov linearization Equation (20). Therefore, if the angular rates are sensed by Equation (20), the twice periodic components in the results should be filtered out by the notch filters G(s), which can be designed as follow:

$$G\left(s\right)=\frac{s^{2}+2\pi as+{\left(2\pi f\right)}^{2}}{s^{2}+2\pi bs+{\left(2\pi f\right)}^{2}}$$

where *f* is the center frequency of the notch filter and given by $f=2f_{motor}=50$ Hz; here, $f_{motor}$ represents the spin frequency of the motor; the parameters *a* and *b* are given by 0.1 and 60, respectively.

The key parameters in the simulation are given in Table 1. Besides, the initial attitude angular velocity of the spacecraft $\omega_{b}=\left[\omega_{bx};\omega_{by};\omega_{bz}\right]=\left[0.001;0.001;0\right]$ rad/s, and ideally, we assume there exists no disturbance torque acting on the spacecraft, but the control torques from GW.

In the following simulation, the static and dynamic measurements are studied, respectively. In both cases, the proposed measurement Equations (20) and (28) will be analyzed.

### 5.2. Static Measurement Validation

**Figure 5:**
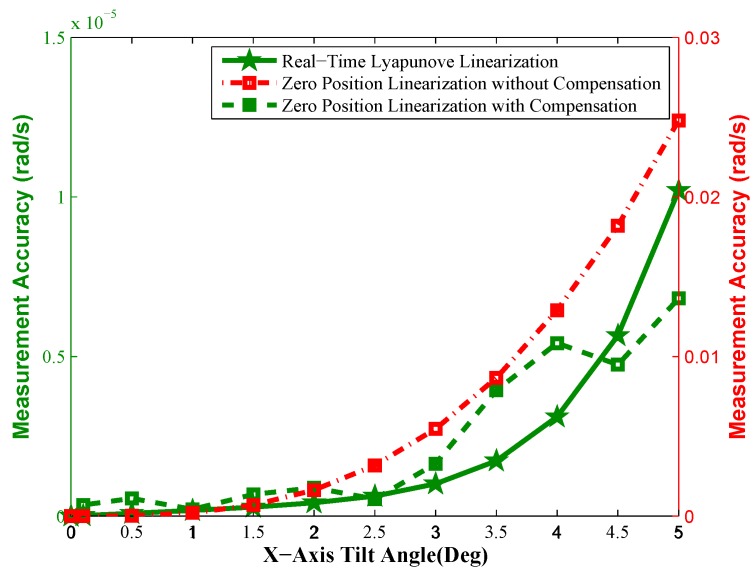
The change of measurement accuracy with the tilt angle.

Considering the situation of the static measurement, which means that there is no radial control torque outputs in this case, the proposed measurement Equations (20) and (28) are applied to measure the spacecraft angular rates at the operating tilt angle range. As an example, when the constant motor speed of 157.04 rad/s is given and the X-axis tilt control commands of the rotor ($\phi_{xd}$) are given by 0,0.1,0.5,1,1.5,2,2.5,3,3.5,4,4.5,5 in turn, the change curves of the measurement errors of Equations (20) and (28) with the tilt angles are shown in Figure 5.

**Figure 6:**
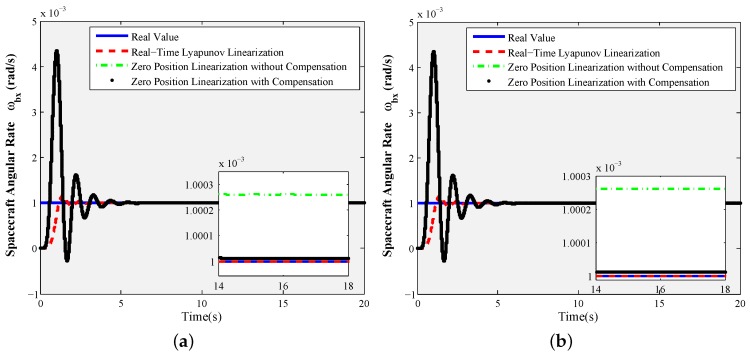
Static measurement $\phi_{xd}=0.1{}^{\circ},\phi_{yd}=0{}^{\circ}$, constant motor spin rate ${\dot{\theta }}_{zd}=157.04$ rad/s: (**a**) X-axis angular rate sensing of the spacecraft $\omega_{bx}$; (**b**) Y-axis angular rate sensing of the spacecraft $\omega_{by}$.

**Figure 7:**
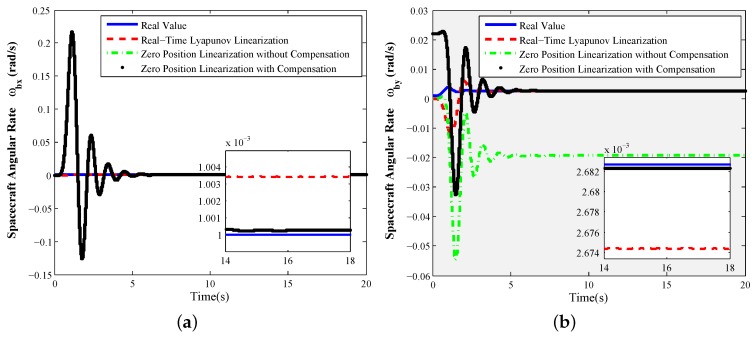
Static measurement $\phi_{xd}=4.8{}^{\circ},\phi_{yd}=0{}^{\circ}$, constant motor spin rate ${\dot{\theta }}_{zd}=157.04$ rad/s: (**a**) *X*-axis angular rate sensing of spacecraft $\omega_{bx}$; (**b**) *Y*-axis angular rate sensing of spacecraft $\omega_{by}$.

From Figure 5, we find:

1. The measurement accuracy decreases with the increase of the tilt angle for both the real-time linearization equation and the zero position linearization equation; however, due to the linearization operating points at zero tilt angles, the measurement accuracy of the latter deteriorates more seriously than the former.
2. The absolute measurement accuracy of the real-time linearization Equation (20) within operating ranges meets the adequate accuracy requirement. Therefore, Equation (20) can be applied to the operating state at arbitrary tilt angles, but if there is not any linearization error compensation for Equation (28), the zero position linearization Equation (28) is more suitable for the small tilt angle of the rotor, rather than the larger tilt angle of the rotor (for example, see Figure 6 and Figure 7.

Further, considering the measurement Equation (28) without the need for sensing the motor rotation angle $\theta_{z}$ and that the fault-tolerant performance of the measurement Equation (28) is more excellent than Equation (20), if the measurement errors of Equation (28) can be compensated, it will be a better choice in the time-sharing multiplexing scheme. Since the linearization errors vary regularly with the tilt angle according to the error curve in Figure 5, the error compensation expressions of the measured spacecraft angular rates ($\omega_{bxe},\omega_{bye}$) are established for Equation (28) based on the simulation error data with polynomial fitting functions, which are given by:

(29) $$\begin{array}{cc} \omega_{bxe}= & -1.447\times 10^{-7}\cdot \phi_{xd}^{5}+1.379\times 10^{-6}\cdot \phi_{xd}^{4}-6.801\times 10^{-6}\cdot \phi_{xd}^{3}\\ & +1.080\times 10^{-5}\cdot \phi_{xd}^{2}-8.840\times 10^{-6}\cdot \phi_{xd}+5.349\times 10^{-7}\\ \omega_{bye}= & -4.355\times 10^{-7}\cdot \phi_{xd}^{5}+1.501\times 10^{-6}\cdot \phi_{xd}^{4}+2.008\times 10^{-4}\cdot \phi_{xd}^{3}\\ & +6.730\times 10^{-6}\cdot \phi_{xd}^{2}-8.229\times 10^{-6}\cdot \phi_{xd}+8.113\times 10^{-8} \end{array}$$

With Equation (29) as the compensation equations of the linearization errors, that is $\omega_{bx\_new}=\omega_{bx}+\omega_{bxe},\;\omega_{by\_new}=\omega_{by}+\omega_{bye}$, then the measurement error curves with the compensated small tilt linearization equations are also shown in Figure 5 and expressed by the green dotted line and squire markers. From this figure, the measurement accuracy with the measurement Equation (28) after the polynomial compensation is improved significantly, even superior to the real-time linearization measurement Equation (20) in this case. Therefore, when the sharing time multiplexing measurement scheme is adopted, compared to Equation (20), the measurement Equation (28) with regular error compensation components is an ideal method of spacecraft angular rate sensing.

Especially, the response curves of the X-axis tilt control commands given by 0.1° and 4.8° are shown as Figure 6 and Figure 7, respectively. From Figure 6, when the rotor is working at the tilt angle of 0.1°, the measurement accuracy of the zero position linearization Equations (28) with compensation terms ($\omega_{bxe},\omega_{bye}$) is improved to precede $10^{-7}$ rad/s compared to the ones without any compensation term. At this point, the real-time Lyapunov linearization measurement can also achieve the perfect measurement performance of the spacecraft angular rates. When the tilt angle of the rotor is increased to 4.8° in Figure 7, the measurement errors of the zero position linearization Equation (28) with compensation terms ($\omega_{bxe},\omega_{bye}$) are far less than $10^{-6}$ rad/s. The real-time Lyapunov linearization measurement accuracy can arrive at $10^{-6}$ rad/s. However, the measurement accuracy of the zero position linearization Equation (28) without any compensation term deteriorates seriously, so that the uncompensated zero position linearization equations are not suitable for the angular rate sensing at the large tilt angle. However, since the motor spin angle sensor needs to be introduced to realize the measurement of the motor spin angle when the real-time Lyapunov linearization measurement equations are used to realize the angular rate sensing, it will make the measurement realization more complex in the time-sharing multiplexing scheme.

Comparing Figure 6 to Figure 7, the foregoing analysis for static measurement can be validated; the most important and useful information is given: zero tilt angle linearization Equation (28) with the regular error compensation components is the best choice for the time-sharing multiplexing scheme of GW.

### 5.3. Dynamic Measurement Validation

**Figure 8:**
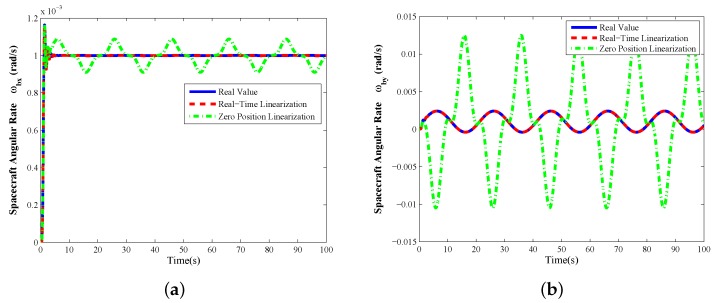
Dynamic measurement $\phi_{xd}=4\sin(2\pi \cdot 0.05t){}^{\circ},\phi_{yd}=0{}^{\circ}$, constant motor spin rate ${\dot{\theta }}_{zd}=157.04+20\sin\left(2\pi \cdot 0.02t\right)$rad/s: (**a**) *X*-axis angular rate sensing of spacecraft $\omega_{bx}$; (**b**) *Y*-axis angular rate sensing of spacecraft $\omega_{by}$.

In this subsection, we investigate the dynamic measurement of the spacecraft angular rates with the GW, in which case, the control command of the X-axis tilt angle $\phi_{xd}$ keeps as time varying and is given by $\phi_{xd}=4\sin\left(2\pi \cdot 0.05t\right)$°, so that the Y-axis attitude angle of the spacecraft can be driven by the gyroscopic moment from the GW. The response curves of the dynamic measurement of the spacecraft angular rates with the GW are shown in Figure 8.

Similarly with the previous static measurement case, in Figure 8, the *X*-axis and *Y*-axis angular rates can be sensed accurately by the real-time Lyapunov’s linearization method using GW, and the measurement equations linearized at zero tilt position no longer satisfy the measurement requirement. Moreover, since the linearization errors depend on the tilt angular rate of the rotor, it is difficult to establish the compensation polynomial equations by simulation data in dynamic measurement. Therefore, even if in the dynamic measurement case, which means that the gyroscopic moments are produced to control the spacecraft attitudes by tilting the rotor of GW along the radial direction, the proposed innovative measurement Equation (20) based on real-time Lyapunov’s linearization is also an effective way.

## 6. Conclusions

The GW can not only realize the function of the two-dimensional angular rate sensing, but also the three-dimensional torque output at the same time. The angular rate sensing with GW could be an effective supplement to the conventional ACS rate gyro configurations.

To achieve the above-mentioned goal, two principal contributions to the angular rate measurement with GW are made in this paper:

(1) By combining the real-time Lyapunov’s linearization with the complex quantity transform, two different measurement models of the spacecraft angular rate sensing with GW are established; we named them as the “real-time Lyapunov linearization measurement model” and the “small tilt measurement model”, respectively.
(2) For both established measurement models of GW, two different application schemes are proposed: “time-sharing multiplexing of actuator and sensor” and “simultaneous realization of actuator and sensor”. Additionally, the advantage and disadvantage of the two measurement schemes is also presented.

Limited to the experiment conditions, simulations are performed instead of the validation experiment with the GW prototype in this paper. By combining the ground calibration, the proposed measurement method needs to be investigated further in the future.

## Figures and Tables

**Table 1:** Gyrowheel and spacecraft design parameter in simulation.

Parameter Name	Value
Rotor transverse-axis inertia $I_{rt}$	$3.458\times 10^{-3}$ kg · m${}^{2}$
Rotor spin-axis inertia $I_{rs}$	$6.402\times 10^{-3}$ kg · m${}^{2}$
Gimbal transverse-axis inertia $I_{gt}$	$1.2758\times 10^{-5}$ kg · m${}^{2}$
Gimbal spin-axis inertia $I_{gs}$	$1.8047\times 10^{-5}$ kg · m${}^{2}$
Torsion spring stiffness $k_{x},k_{y}$	0.092Nm/rad
Torsion spring damping $c_{x},c_{y}$	0 Nm/(rad/s)
Operating tilt range of rotor $\phi_{x},\phi_{y}$	$0{}^{\circ}\leq \phi_{x},\phi_{y}\leq 5{}^{\circ}$
Spacecraft inertia $I_{\mathrm{sat}}$	$diag\left[\begin{array}{ccc} 50 & 50 & 50 \end{array}\right]$ kg · m${}^{2}$

## Abbreviations

The following abbreviations are used in this manuscript:

GW: Gyrowheel
ACS: Attitude control system
DTG: Dynamically-tuned gyroscope
VSCMG: Variable-speed control moment gyroscope
CMG: Control moment gyroscope
MSDGCMG: Magnetically-suspended double-gimbal control moment gyroscope
AMBs: Active magnetic bearings
